# EMERGENCY DEPARTMENT UTILIZATION BY SPINA BIFIDA PATIENTS IN FLORIDA 2016–2020

**DOI:** 10.2340/jrm.v57.41412

**Published:** 2025-03-09

**Authors:** Sarah WHITTEKER, Dhyey DESAI, Hannah BAKER, Sudarshan SRIRANGAPATANAM, Lucas R. WIEGAND, Hubert S. SWANA

**Affiliations:** 1 Florida State University, College of Medicine, Tallahassee; 2 University of Central Florida, College of Medicine, Orlando; 3 Florida International University, Herbert Wertheim College of Medicine, Miami; 4 Orlando Health/Arnold Palmer Hospital, Orlando, FL, USA

**Keywords:** spina bifida, emergency department utilization, transitional care, spinal dysraphism

## Abstract

**Objective:**

To investigate emergency department use of spina bifida patients in Florida and identify presenting diagnoses across all age groups.

**Design:**

Retrospective cohort study.

**Subjects/Patients:**

Individuals with a diagnosis of spina bifida who presented to the emergency department between 2016 and 2020 in Florida.

**Methods:**

The State Emergency Department Databases of Florida from the Healthcare Cost and Utilization Project were utilized. Principal diagnosis codes were queried, and patients were classified into paediatric (ages 0–19), transitional (ages 20–29), adults (ages 30–59), and geriatric (ages 60 or greater). To analyse patient-level factors and observed case numbers, χ^2^ testing was used. The transitional period was further evaluated by pair-wise tests of proportions with Bonferroni adjustment.

**Results:**

The transitional age group (20–29) patients had the highest number of emergency department encounters (24.1%). The most common presenting diagnoses were infections (23.1%) followed by epilepsy and seizure (17.3%). Transitional groups were responsible for the most emergency department encounters across all categories (*p* < 0.001).

**Conclusion:**

Transitional ages patients (20–29) were responsible for the majority of encounters, suggesting the significance and the need for continued multidisciplinary coordinated care during the transitional of care between paediatric and adult settings for patients with spina bifida.

## LAY ABSTRACT

Spina bifida is a congenital condition in which a patient’s spinal column does not close properly before birth, leading to a significant effect on movement, sensation, and organ function. Due to the involvement of multiple organ systems, patients require specialized medical care throughout their lives. In the United States, most paediatric patients can receive care in multidisciplinary clinics or paediatric hospitals. Unfortunately, adult patients with spina bifida often encounter challenges accessing such care. This can lead to an increased reliance on the emergency department for conditions that otherwise may be treated in a primary care setting or even prevented. This study aimed to evaluate emergency department utilization by spina bifida patients in Florida and to investigate the common presenting diagnoses across their lifetime. Many visits were for preventable conditions. This highlights the need for access to quality primary and specialty care across all ages, especially during the transition of care.

Spina bifida (SB) is a common congenital anomaly, affecting approximately 3 in every 10,000 births worldwide, 1 in every 2,758 births in the United States, and 2.8 in every 10,000 births in Florida (1–3). In SB occulta, the mildest form of SB, one or more vertebrae are missing, but there is no opening or fluid sac, and it is covered by skin (4). SB occulta is usually asymptomatic and found incidentally, with symptoms of spinal cord tethering found in about 1 in 1,000 cases after a growth spurt during adolescence (4–5), In closed neural tube defects of SB, there are malformations of fat, bone, or meninges (4). Failure of neural tube closure during the first trimester of gestation results in inappropriate exposure of the posterior elements of the spine including meninges and cerebrospinal fluid, termed meningocele, or meninges, cerebrospinal fluid, and spinal cord, known as myelomeningocele. Medical complications are variable and arise from a dysfunctional nervous system to include epilepsy and seizure, headache, hydrocephalus, bowel and bladder dysfunction, and mobility challenges. Infections of the urinary tract, meningitis, pneumonia, and skin infections can also occur. Given the widespread multi-system effects, most SB patients would benefit from lifelong coordinated, multidisciplinary care. Such disciplines include, but are not limited to, neurosurgery, physical therapy, urology, social work, orthopaedics, nutrition, paediatrics, and physiatry (6–7). Historically, patients with SB rarely survived past the teenage years, but due to specialized multidisciplinary SB care and medical advancements, most patients can now reach adulthood (8).

Although there is an emphasis on the multidisciplinary model of care in a paediatric setting, this level of support is not readily available for adult patients (7). The transitional period from paediatric to adult care for SB patients is usually between 16 and 21 years old, and there is a large variation with regard to when or if transition occurs. Without the necessary resources to provide comprehensive care, adult SB patients can be left without appropriate specialty services, shifting the burden of disease management to the primary care provider (7). Lack of primary care services for adult patients can exacerbate this problem, as they often serve an additional role of specialty care coordinator. It has been reported that less than 25% of adults with SB had a primary care provider (9). This lack of access to primary and specialty care can lead to over-utilization and reliance on emergency services (10).

Patients with SB have been found to utilize emergency services more frequently, incurring greater healthcare costs and decreasing their health-related quality of life (11–12). The objective of this study was to examine emergency department (ED) utilization for patients with SB in Florida and elucidate the most common presenting diagnoses across their lifespan.

## METHODS

### Data source

The State Emergency Department Databases (SEDD) for the state of Florida from the Healthcare Cost and Utilization Project (HCUP) between 2016 and 2020 were utilized. Data elements available included patient-level demographics such as age, gender, race, ethnicity, and insurance status, and diagnosis codes in the form of International Classification of Diseases version 10 (ICD-10) codes. All patient identifiers were de-identified at source and the project was deemed non-human subjects research by the institutional review board (IRB).

### Inclusion and exclusion

All patients regardless of age who had any diagnosis or history of diagnosis of SB (Appendix SI) across all state datasets including inpatient, ED, and ambulatory surgery were included as the study population. Next, all ambulatory surgery and inpatient encounters were excluded to restrict the dataset to a population of patients with SB who presented to the ED over a 5-year period.

### Presenting diagnosis

Principal diagnosis codes were systematically collected for all ED encounters per HCUP specifications and were available in the form of ICD-10 codes. The principal diagnosis was queried and categorized as infections (cellulitis, ulcers, sepsis, and -osteomyelitis), epilepsy and seizure, bowel dysfunction, -cystitis, headache, pyelonephritis, fluid and electrolyte imbalance, pneumonia, urinary retention, hydrocephalus/shunt malfunction, and urinary incontinence (see Appendix SI). In our analysis, cellulitis consisted of soft tissue infection with intact skin.

### Statistical analysis

Patient ages were stratified into decades of life beginning from birth to age 60 and greater. They were further classified into paediatric (ages 0–19), transitional (ages 20–29), adults (ages 30–59), and geriatric (ages 60 or greater). Patient-level factors associated with the distribution of ED utilization by age were analysed using a χ^2^ test with a significance level of 0.001. Common SB-related presenting diagnoses were evaluated using visit data and their corresponding principal diagnosis. The significance of observed case numbers was evaluated with a χ^2^ test with a significance level of 0.001. The transitional period was evaluated by first analysing the observed number of cases against the expected number of cases and performing pair-wise tests of proportions with Holm adjustment for multiple testing correction.

## RESULTS

Between 2016 and 2020, 4,987 unique SB patients presented to an ED in Florida, totalling 35,682 encounters. The majority of encounters occurred among those who identified as white (62.6%), female (63.2%), and between 20 and 29 years of age (24.1%). Medicaid was the most common insurance status with 40.8% utilization (Table I).

**Table I:** Demographic characteristics

Factor	Paediatric		Transitional	Adult			Geriatric	*p*-value
Age groups (years)	0–9, *n* = 2,490	10–19, *n* = 3,832	20–29, *n* = 8,611	30–39, *n* = 8,460	40–49, *n* = 5,856	50–59, *n* = 3,430	60+, *n* = 3,003	
Sex								< 0.001
Male	1,265 (50.80%)	1,609 (41.99%)	2,785 (32.34%)	2,976 (35.18%)	1,851 (31.16%)	1,439 (41.95%)	1,194 (39.76%)	
Female	1,225 (49.20%)	2,223 (58.04%)	5,826 (67.66%)	5,484 (64.82%)	4,005 (68.39%)	1,991 (58.05%)	1,809 (60.24%)	
Race								< 0.001
White	1,120 (44.98%)	1,738 (45.38%)	4,984 (57.88%)	5,603 (66.23%)	4,023 (68.70%)	2,420 (70.55%)	2,454 (81.72%)	
Black	496 (19.92%)	728 (19.01%)	1,367 (15.88%)	1,169 (13.82%)	825 (14.09%)	409 (11.92%)	161 (5.36%)	
Hispanic	777 (30.92%)	1,178 (30.76%)	2,102 (24.41%)	1,555 (18.38%)	943 (16.10%)	549 (16.01%)	324 (10.79%)	
Other	97 (3.86%)	188 (4.92%)	158 (1.83%)	133 (1.34%)	65 (1.11%)	52 (1.50%)	64 (2.13%)	
Insurance								< 0.001
Medicare	6 (0.24%)	5 (0.13%)	961 (11.16%)	2,053 (24.27%)	2,322 (39.65%)	1,538 (44.84%)	2,550 (84.92%)	
Medicaid	1,938 (77.83%)	2,802 (73.12%)	4,079 (47.37%)	3,416 (40.38%)	1,535 (26.21%)	662 (19.30%)	140 (4.66%)	
Private	325 (13.05%)	731 (19.08%)	1,972 (22.90%)	1,642 (19.41%)	977 (16.68%)	733 (21.37%)	228 (7.59%)	
Self-pay	35 (1.41%)	111 (2.90%)	1,294 (15.03%)	996 (11.78%)	648 (11.07%)	309 (9.01%)	42 (1.40%)	
Other	186 (7.46%)	183 (4.78%)	305 (3.51%)	353 (4.17%)	374 (6.39%)	188 (5.43%)	43 (1.43%)	

The most common presenting diagnoses were infections, including cellulitis, ulcers, sepsis, and osteomyelitis (2.3%), followed by epilepsy and seizure (1.7%). Bowel dysfunction and cystitis were also frequent presentations, accounting for 1.2% and 1.1% of total encounters respectively. Less common were headache (1.0%), pyelonephritis (0.7%), fluid and electrolyte imbalance (0.6%), and pneumonia (0.5%). Bladder dysfunction and hydrocephalus/shunt malfunction were the 2 least reported diagnoses contributing to 0.4% and 0.2% of encounters respectively (Table II).

**Table II:** Number of encounters

Factor	Paediatric		Transitional	Adult			Geriatric	*p*-value
Age groups (years)	0–9, *n* = 2,490	10–19, *n* = 3,832	20–29, *n* = 8,611	30–39, *n* = 8,460	40–49, *n* = 5,856	50–59, *n* = 3,430	60+ *n* = 3,003	
ED diagnosis^1^								
Infections^2^ (*n* = 808)	56 (6.93%)	98 (12.13%)	164 (20.30%)	192 (23.76%)	153 (18.94%)	92 (11.39%)	53 (6.56%)	< 0.001
Cellulitis (*n* = 551)	44 (7.99%)	58 (10.53%)	105 (19.06%)	124 (22.50%)	113 (20.51%)	65 (11.80%)	42 (7.62%)	
Ulcers (*n* = 197)	8 (4.06%)	33 (16.75%)	47 (23.86%)	54 (27.41%)	23 (11.68%)	24 (12.18%)	8 (4.06%)	
Sepsis (*n* = 43)	3 (6.98%)	7 (16.28%)	7 (16.28%)	11 (25.58%)	11 (25.58%)	2 (4.65%)	2 (4.65%)	
Osteomyelitis (*n* = 17)	1 (5.88%)	0 (0.00%)	5 (29.41%)	3 (17.65%)	6 (35.29%)	1 (5.88%)	1 (5.88%)	
Epilepsy and seizure (*n* = 604)	116 (19.21%)	87 (14.40%)	159 (26.32%)	96 (15.89%)	77 (12.75%)	39 (6.46%)	30 (4.97%)	< 0.001
Bowel dysfunction (*n* = 444)	82 (18.47%)	98 (22.07%)	69 (15.54%)	89 (20.05%)	56 (12.61%)	21 (4.73%)	29 (6.53%)	< 0.001
Cystitis (*n* = 402)	23 (5.72%)	51 (12.69%)	112 (27.86%)	85 (21.14%)	84 (20.90%)	19 (4.73%)	28 (6.97%)	< 0.001
Headache (*n* = 345)	3 (0.87%)	39 (11.30%)	103 (29.86%)	100 (28.99%)	58 (16.81%)	34 (9.86%)	8 (2.32%)	< 0.001
Pyelonephritis (*n* = 266)	15 (5.64%)	32 (12.03%)	74 (27.82%)	65 (24.44%)	54 (20.30%)	18 (6.77%)	8 (3.00%)	< 0.001
Fluid and electrolyte imbalance (*n* = 213)	17 (7.98%)	19 (8.92%)	49 (23.00%)	41 (19.25%)	27 (12.68%)	30 (14.08%)	30 (14.08%)	< 0.001
Pneumonia (*n* = 175)	20 (11.43%)	30 (17.14%)	36 (20.57%)	29 (16.57%)	11 (6.29%)	25 (14.29%)	24 (13.71%)	0.02
Bladder dysfunction (*n* = 155)	6 (3.87%)	14 (9.03%)	44 (28.39%)	32 (20.65%)	21 (13.55%)	20 (12.90%)	18 (11.61%)	< 0.001
Hydrocephalus/shunt (*n* = 80)	15 (18.75%)	15 (18.75%)	20 (25.00%)	13 (16.25%)	15 (18.75%)	2 (2.50%)	0 (0.00%)	< 0.001

1 All percentages for these are row percentages.

2 The rate of Infection is equal to the sum of cellulitis, ulcers, and osteomyelitis.

**Fig. 1:**
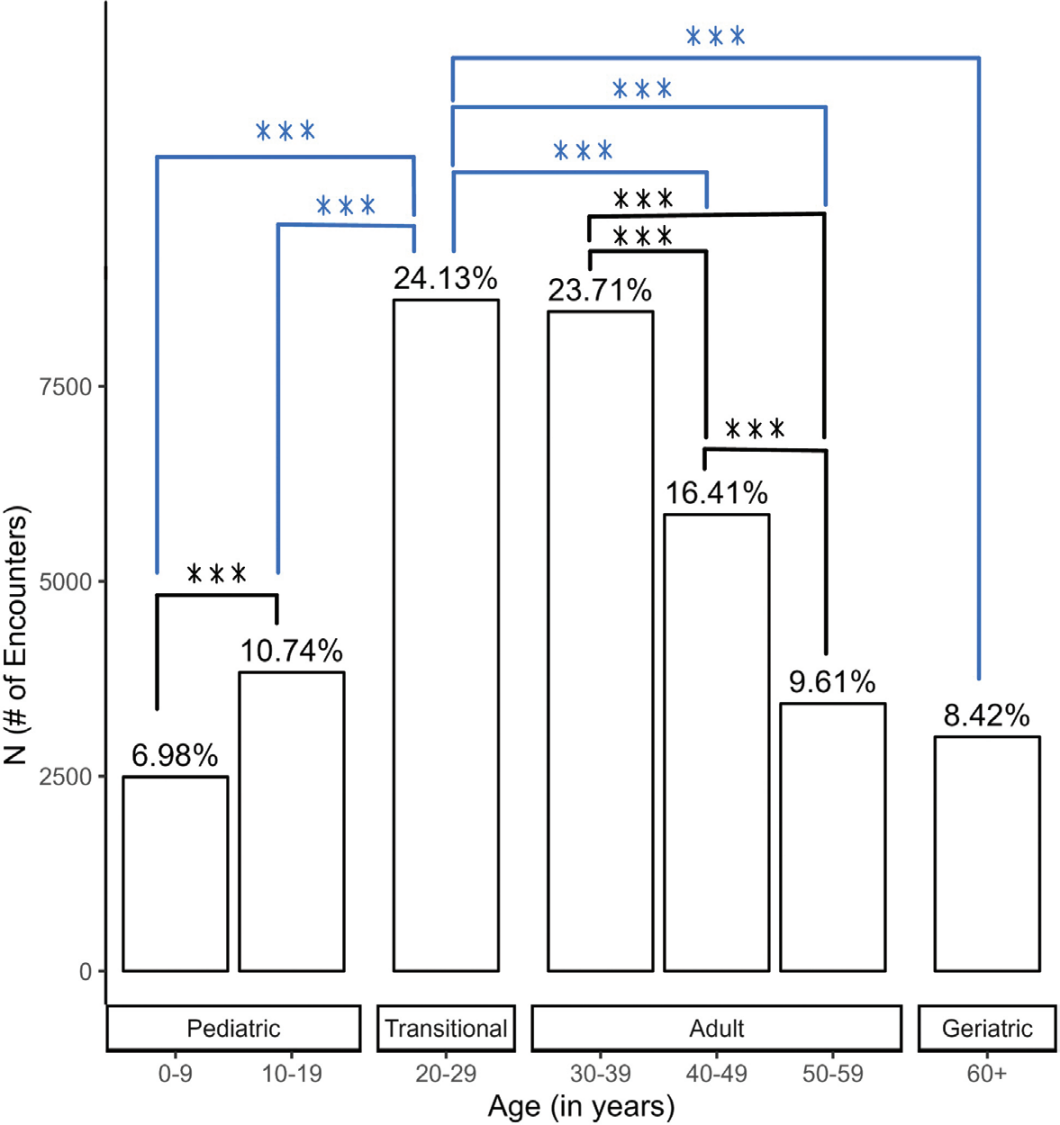
**Encounter frequency across age groups.** A total of 35,682 Emergency Department encounters from 4,987 unique patients were identified. The highest encounter rate (24.13%) occurred among individuals aged 20–29 years, while children aged 0–9 years visited least frequently (6.98%). With Holm adjustment, data were significant (***p < 0.001) between intra-group evaluations and evaluations of each age group to the transitional group.

**Fig. 2:**
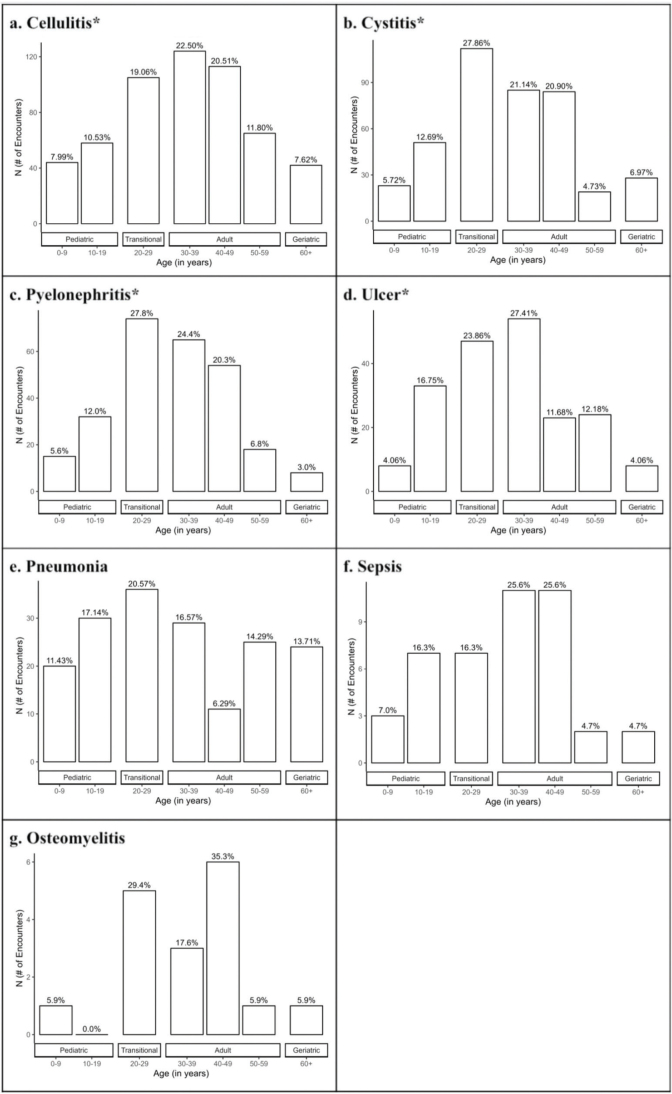
**Number of infection related encounters by age.** Infection-related complications include: (a) cellulitis, (b) cystitis, (b) pyelonephritis, (d) ulcer, (e) pneumonia, (f) sepsis, and (g) osteomyelitis. The data were significant (*p < 0.001) across the age groups for cellulitis, cystitis, pyelonephritis, and ulcer.

**Fig. 3:**
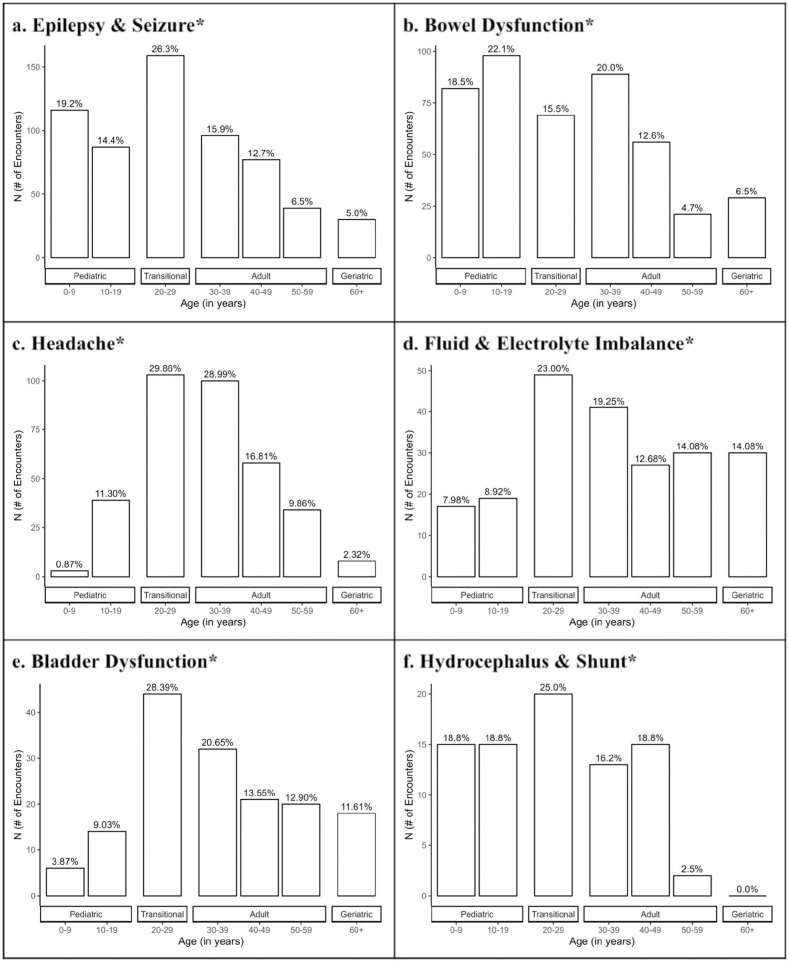
**Number of non-infection related encounters by age.** Non-infection complications include: (a) epilepsy and seizure, (b) bowel dysfunction, (c) headache, (d) fluid and electrolyte imbalance, (e) bladder dysfunction, and (f) hydrocephalus and shunt. The data were significant (p* < 0.001) across the age groups for all of the above complications.

Transitional patients (ages 20–29) had the highest number of ED encounters (24.1%), and paediatric patients (ages 0–9) visited the least (7.0%) (Fig. 1). Infectious presentations including cellulitis, cystitis, pyelonephritis, and ulcers showed significant differences (*p* < 0.001) across age groups. Adult patients (ages 30–39) were more likely to be affected by cellulitis (22.5%) and ulcers (27.4%), while transitional patients (ages 20–29) were more likely to present with cystitis (27.9%) and pyelonephritis (27.8%) (Fig. 2). Non-infectious presentations including epilepsy and seizure, bowel dysfunction, headache, fluid and electrolyte imbalance, bladder dysfunction, and hydrocephalus and shunt showed significant differences (*p* < 0.001) across age groups. Transitional patients (ages 20–29) were responsible for the most encounters within all categories (*p* < 0.001) (Fig. 3).

## DISCUSSION

Adults with SB continue to face significant health challenges that require coordinated specialty care. The variation in timing and implementation of transition education, combined with the fact that less than 25% of adults with SB have a primary care provider, highlights a major gap in care for patients with SB. Transitional patients (ages 20–29) presented to the ED most often (*n* = 8,611, 24.13%), followed by adult patients (ages 30–39) (*n* = 8,460, 23.71%). These age groups represent when patients typically transition to, and should subsequently receive, adult medical care (13). Further analysis of specific ED presenting diagnoses for SB patients revealed that infections, followed by neurological conditions (epilepsy and seizure), and bowel dysfunction were the most common presenting diagnoses (*n* = 1,856, 5.20%). Bladder dysfunction and hydrocephalus/shunt malfunction were the least in frequency (*n* = 235, 0.66%). While infectious complications such as cellulitis, cystitis, pyelonephritis, and ulcers affected both transitional-aged patients and adults, non-infectious complications burdened the transitional-age group the most compared with paediatric and adult populations. This suggests that many of these patients could have been treated in the outpatient primary and/or specialty care setting(s).

This study included 2,342 patients identifying as White, 51,555 as Black, 7,428 as Hispanic, and 757 as Other. Patients identifying as Black and Hispanic presented to the ED most often during the transitional years (ages 20–29). The nature of American healthcare is one possible explanation for the insurance analysis results in Table I. At a young age, many rely on Medicaid and private insurance through their families. The number of self-paid patients rises as the patients transition to adulthood and are no longer covered by family insurance and, as patients continue to age, Medicaid enrolment increases.

In a study of the general population, Fortuna and colleagues noted that young adults (20–29 yrs) most commonly presented to the ED for trauma, GI issues, or musculoskeletal pain (10). Aside from traumatic injury, these presentations also reflect treatment that could likely be managed in an outpatient setting. Our study found that young adults with SB present with different diagnoses to the ED than the general population. This information is useful when designing primary, specialty, and preventative care programmes for SB patients.

Spina bifida patients require comprehensive care at all stages of life including paediatric, transitional, and adult settings given the lifelong nature of the disease, and advancements in medical care that have made such life expectancy possible. Previous literature, however, has identified the lack of established adult care by many patients, leading to increased utilization of ED services for preventable health conditions (13–14). This may result from the lack of established adult centres for SB care. According to the Spina Bifida Association, 109 clinics within the United States treat paediatrics and only 32 clinics treat adults as of August 2024. Furthermore, there are zero multidisciplinary clinics for adults in Florida, with the closest adult clinic in Birmingham, Alabama (15).

However, 2 district models for adult care have been developed in Florida. The first model, in Orlando, FL, operates as a provider network where specialists consult with patients individually. This approach can pose challenges to care coordination. The second model, successfully implemented in Jacksonville, FL, is the Jacksonville Health and Transition Services (JaxHATS) clinic, a medical home serving individuals aged 16 to 26 with chronic medical conditions, including SB. A survey of patients receiving care at JaxHATS reported ease in regular medical care visits, with few ED visits for SB-related conditions. Most patients reported a good quality of life (16). This suggests that patients of transitional age would benefit from a multidisciplinary clinic setting such as JaxHATS. Larger studies are needed to further evaluate the positive effect of medical homes for this population (16).

Previous studies in SB patients grouped into children, adults who transitioned to adult care, and adults who did not transition to adult care demonstrated that adults who did not transition their adult care had greater ED utilization, inpatient admissions, and surgeries. Young adults (20–24 years old) had more ED visits than adolescents (15–19 years old), with SB patients discharged from paediatric care without follow-up being more likely to visit the ED (*p* = 0.03) (11, 17, 18). Hospital admission -rates were 19.4 times higher in youth and 12.4 -times higher in adult patients with SB compared with the general population (8). Increased utilization is multifactorial and attributed to increased baseline need, socioeconomic factors affecting access to adult care resulting in ED services used as primary care, and lack of coordinated care from teenage to adult years (18–19). There were similarities in ambulation status, insurance, and distance of residence from the clinic amongst those who completed follow-up and those who did not, suggesting other factors hinder the establishment of adult care. Reasons for failure to transition to adult care include patient-reported lack of concern to find a provider due to current asymptomatic status, fatigue from frequent healthcare, and poor physician–patient communication (20). Improved patient education on the chronic nature and associated risks of their condition with insufficient follow-up is essential. Healthcare providers should ensure structured discharge from paediatric services with confirmed future adult care to reduce preventable ED visits.

Early mortality in patients with spina bifida can be due to multiple medical causes. Urinary tract infections and meningitis can lead to sepsis and death (21). Recurrent urinary tract infections, obstruction, and elevated bladder pressures can lead to renal failure (22). Decreased mobility, restrictive lung disease, and sleep-disordered breathing in SB patients can lead to oxygen dependence, pneumonia, and possibly sudden death (23, 24). Obesity and diabetes can also contribute to cardiovascular disease (25). Hydrocephalus, a common occurrence in SB patients, can lead to increased intracranial pressures, seizures, and death (26, 27). Individuals with spina bifida and their caregivers face significant social, psychological, and economic burdens. This lack of access to health care results in decreased preventative care and an increased reliance on emergency services (28, 29). The increased ED utilization among the transitional aged patients (ages 20–29) can be a reflection of early mortality, resulting in the relatively decreased utilization among paediatric patients (ages 0–19) observed. However, the trends continue into adulthood, which shows that transitional groups (ages 20–29) still present the potential for intervention to aid in care of those who survive paediatric ages.

Chronic complications in SB not only add to the complexity of adult care, but may also adversely affect patients’ quality of life (30). Children and adolescents aged 8–15 years old with SB reported significantly lower health-related quality of life (HRQoL) across all domains including physical, psychosocial, emotional, social, and school at both 8–15 years and 10–17 years compared with individuals without SB (12). As this trend may continue into adulthood, it is crucial to enhance both medical care and quality of life (12).

### Limitations

Limitations of our study include the retrospective nature of data collection. Additionally, due to the nature of the dataset, we rely on accurate ICD code classification and associated billing to draw conclusions on presenting ED diagnoses. We do not have access to individual patient medical history and additional ED visit details, which could lend further information to the root cause of the encounter. Further studies with access to this information would add to the strength of established conclusions. Despite these limitations, this study highlights trends and measures at the population level in the state of Florida which can be used to track changes in measures as new policies and multidisciplinary care centres are implemented.

### Conclusion

Emergency department encounters in Florida for patients with spina bifida include conditions such as infection, bowel dysfunction, and cystitis, which are potentially preventable conditions when managed in an outpatient setting. Notably, the majority of these encounters were from young adult patients aged 20–29. These findings highlight the impact of transitional care, or lack thereof, between paediatric and adult services for patients with spina bifida and the continued need for multidisciplinary care in adulthood.

## Supplementary Material


