# Supplementary material for: EMERGENCY DEPARTMENT UTILIZATION BY SPINA BIFIDA PATIENTS IN FLORIDA 2016–2020
Source: J Rehabil Med. 2025 Mar 9;57:41412. doi: 10.2340/jrm.v57.41412 (PMC11971943; doi:10.2340/jrm.v57.41412)
Supplement: Supplementary file 1 [file JRM-57-41412-s1.pdf]

Supplementary material has been published as submitted. It has not been copyedited, or typeset by Journal of Rehabilitation Medicine

Appendix SI

Inclusion Criteria

**Diagnosis**	**ICD-10**
Cervical spina bifida with hydrocephalus	Q05.0
Thoracic spina bifida with hydrocephalus	Q05.1
Lumbar spina bifida with hydrocephalus	Q05.2
Sacral spina bifida with hydrocephalus	Q05.3
Unspecified spina bifida with hydrocephalus	Q05.4
Cervical spina bifida without hydrocephalus	Q05.5
Thoracic spina bifida without hydrocephalus	Q05.6
Lumbar spina bifida without hydrocephalus	Q05.7
Sacral spina bifida without hydrocephalus	Q05.8
Spina bifida, unspecified	Q05.9
Arnold-Chiari syndrome with spina bifida	Q07.01
Arnold-Chiari syndrome with spina bifida and hydrocephalus	Q07.03
Spina bifida occulta	Q76.0

Exclusion Criteria

**Diagnosis**	**ICD-10**
Arnold-Chiari syndrome without spina bifida or hydrocephalus	Q07.00
